# Simultaneous Quantification of Baricitinib and Methotrexate in Rat Plasma by LC-MS/MS: Application to a Pharmacokinetic Study

**DOI:** 10.3797/scipharm.1510-08

**Published:** 2015-12-20

**Authors:** Sridhar Veeraraghavan, Satheeshmanikandan R. S. Thappali, Srikant Viswanadha, Swaroop Vakkalanka, Manivannan Rangaswamy

**Affiliations:** 1 Incozen Therapeutics Pvt. Ltd, Alexandria Knowledge Park, Shamirpet, Hyderabad, 500078, India; 2 CRD, PRIST University, Vallam, Thanjavur 613403. India, India; 3 Dept of Pharmaceutics, Annai JKK Sampoorani Ammal College of Pharmacy, Namakkal - 638 183, India

**Keywords:** Baricitinib, Methotrexate, Plasma, LC-MS/MS, Bioanalytical

## Abstract

Efficacy assessments using a combination of baricitinib and methotrexate necessitate the development of an analytical method for the determination of both drugs in plasma with precision. A high-performance liquid chromatography-tandem mass spectrometry (LC-MS/MS) method was developed for the simultaneous determination of baricitinib and methotrexate in rat plasma. Extraction of baricitinib, methotrexate, and tolbutamide (internal standard; IS) from 50 µL of rat plasma was carried out by protein precipitation with methanol. Chromatographic separation of the analytes was performed on the YMC pack ODS AM (150 mm × 4.6 mm, 5 µm) column under gradient conditions with methanol: 2.0 mM ammonium acetate buffer as the mobile phases at a flow rate of 1 mL/min. The precursor ion and product ion transition for both analytes and IS were monitored on a triple quadrupole mass spectrometer, operated with selective reaction monitoring in positive ionization mode. The method was validated over a concentration range of 0.5–250.00 ng/mL for baricitinib and methotrexate. Mean extraction recoveries for baricitinib, methotrexate, and IS of 86.8%, 89.4%, and 91.8% were consistent across low, medium, and high QC levels, respectively. Precision and accuracy at low, medium, and high quality control levels were less than 15% across the analytes. Benchtop, wet, freeze-thaw, and long-term stability were evaluated for both of the analytes. The analytical method was applied to support the pharmacokinetic study of simultaneous estimation of baricitinib and methotrexate in Wistar rats. Assay reproducibility was demonstrated by reanalysis of 18 incurred samples

## Introduction

Rheumatoid arthritis is a debilitating disease that affects the quality of life and productivity of millions worldwide. This autoimmune disease is characterized by inflammation of the joints that leads to damage of the cartilage if left unchecked [1]. Remission of rheumatoid arthritis is desirable and is based on composite scores of disease activity.

Methotrexate, a disease-modifying anti-rheumatic drug (DMARD), is one of the most commonly prescribed agents in patients with rheumatoid arthritis. This folate analogue works by inhibiting the enzyme dihydrofolate reductase and impacts proliferation of lymphocytes and other cells that cause joint inflammation [2]. Besides its immunesuppressive activity, methotrexate reduces osteoclastogenesis, thereby benefitting patients with high osteoclast activity by inhibiting osteoporosis and associated joint destruction [3]. However, the adverse effects encountered with the use of methotrexate coupled with compliance issues among patients necessitate the need to develop safer therapies that can be administered conveniently by the oral route. With phosphorylation of kinases such as Janus kinase (JAK) emerging as a novel signaling pathway regulating the pathology of rheumatoid arthritis, efforts have been made to discover novel and potent inhibitors of the JAK signaling cascade [4]. The JAK family represents four tyrosine receptor kinases that participate in cytokine receptor signaling with selective JAK inhibitors viewed to have considerable potential as DMARD in rheumatoid arthritis [5]. Baricitinib, a selective inhibitor of JAK-1 and JAK-2, demonstrated efficacy in rodent models of rheumatoid arthritis [6] and is currently undergoing evaluation in the clinic. In a phase 2 randomized trial, baricitinib was well-tolerated and was associated with reduced disease burden in methotrexate-inadequate responders with the active disease [7]. A phase 3 clinical trial evaluating the effect of the combination of baricitinib and methotrexate in rheumatoid arthritis is currently ongoing (NCT01711359).

To the best of our knowledge, the pharmacokinetics, pharmacodynamics, and safety of baricitinib are reported for the quantification of baricitinib in plasma [8]. Methods for the determination of methotrexate in biological fluids by HPLC-UV or LC-MS/MS have been reported [9–12]. However, reports describing a LC-MS/MS-based method for simultaneous determination of baricitinib and methotrexate in plasma are not available. Simultaneous detection of baricitinib and methotrexate in plasma would help establish a pharmacokinetic and pharmacodynamic co-relation in animal models that would require administration of both drugs to achieve maximal efficacy. In the current article, we describe a highly sensitive, selective, and rapid LC-MS/MS method that was developed and fully validated for simultaneous estimation of baricitinib and methotrexate in rat plasma. This method offers a small turnaround time for analysis and utilizes only 50 µL of rat plasma for sample processing using simple protein precipitation extraction. Translation of this methodology to pharmacokinetic studies is also demonstrated by reanalysis of the incurred sample.

**Fig. 1:**
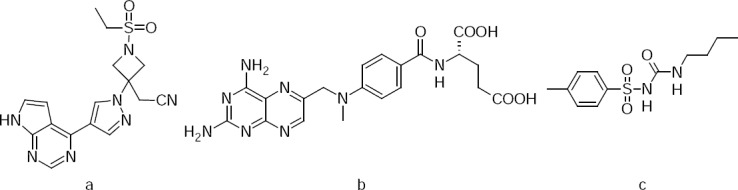
Structures of (a) baricitinib, (b) methotrexate, and (c) tolbutamide.

## Materials and Methods

### Chemical and Reagents

Baricitinib was obtained from EOS Med Chem Co. Ltd, China. Methotrexate, tolbutamide, and ammonium acetate were obtained from Sigma-Aldrich, Germany. Methanol and acetonitrile (HPLC gradient grade) were procured from RCI Lab Scan, Thailand. Ultra pure water of 18 MΩ/cm was obtained from a Milli-Q purification system, Millipore, MA, USA.

### Liquid Chromatographic and Mass Spectrometric Conditions

Reversed-phase chromatographic analysis of analytes was achieved on a Shimadzu SIL 20 AC HT system (Shimadzu Corporation, Japan). Separation of analytes and IS was performed on the YMC Pack ODS AM (150 mm × 4.6 mm, 5 µm) analytical column (YMC®-PACK, JAPAN), maintained at 40°C in a column oven (CTO-10ASVP). Ten microliters of each sample were loaded onto the column, separated, and eluted using a gradient mobile phase consisting of methanol (A): 2 mM ammonium acetate buffer, (B); (minutes, % mobile phase A): (0, 10), (1.5, 70), (4.5, 70), (4.7, 10), (7.5, 10). For gradient elution, the flow rate of the mobile phase was kept at 1.0 mL/min with 70% flow split after post-column elution. Flow was directed to the ion spray interface. Autosampler (SIL20ACHT) temperature was maintained at 10°C. Mass spectrometric detection of analytes and IS was carried out on a triple quadrupole mass spectrometer (Thermo Scientific - Finnigan TSQ Quantum Ultra, San Jose, CA, USA), equipped with heated electrospray ionization and operated in positive ionization mode. Optimized mass parameters and SRM transitions for analytes and IS are given in Table 1. Selective reaction monitoring (SRM) mode was used for data acquisition. Peak integration and calibration were carried out using LC Quan 2.5.2 software (Thermo Scientific).

**Tab. 1:**
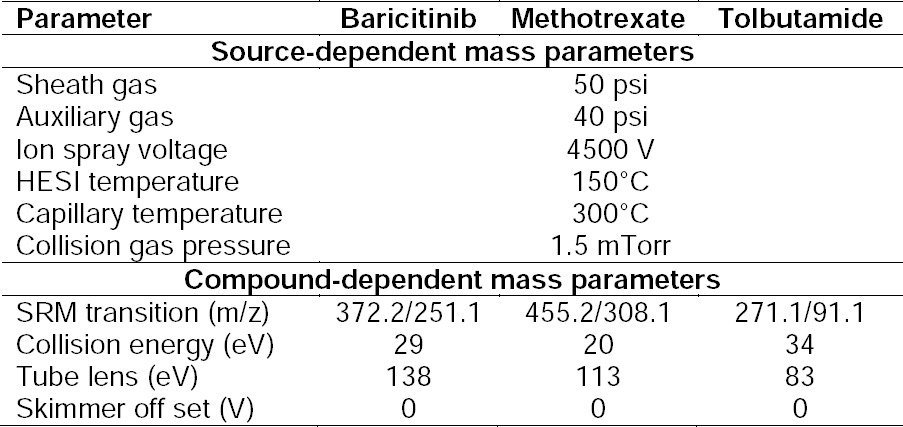
Optimized mass parameters for baricitinib, methotrexate, and tolbutamide

### Calibration Standard and Quality Control Samples

Stock solutions (0.2 mg/mL) of baricitinib, methotrexate, or tolbutamide were prepared by dissolving accurately weighed amounts in acetonitrile. Calibration standards (CSs) and quality control (QC) samples were made by spiking blank plasma with appropriate volumes of working solutions. Final calibration standard concentrations for baricitinib/methotrexate were 0.48/0.49, 0.97/0.98, 1.94/1.96, 3.88/3.92, 7.76/7.85, 15.51/15.69, 31.03/31.38, 62.06/62.77, 124.11/125.54, and 248.22/251.08 ng/mL, respectively. The QC samples were prepared at four concentration levels; 186.17/188.31 ng/mL (HQC, high quality control), 93.08/94.15 ng/mL (MQC, medium quality control), 1.43/1.45 ng/mL (LQC, low quality control), and 0.48/0.49 ng/mL (LLOQ QC, lower limit of quantification quality control), for the baricitinib/methotrexate combination. Tolbutamide (IS) stock solution was diluted with methanol to achieve a final concentration of 125 ng/mL. Standard stock and working solutions were stored at 2–8°C until further use.

### Extraction Procedure

Analytes were extracted from rat plasma by protein precipitation. Briefly, 150 µL of precipitating solution (IS 125 ng/mL) was added to an aliquot of 50 µL plasma and mixed for 3 minutes (IKA vortex, Genius 3). The mixture was centrifuged at 14,000 rpm at 10°C for 5 min. Supernatant (10 µL) was injected into the chromatographic system.

### Validation Procedures

System suitability was determined by injecting six consecutive samples of aqueous standard mixture of analytes and IS at the start of each batch. System performance was assessed by injecting one extracted blank (without analytes and IS) and one extracted LLOQ sample with IS at the beginning of each analytical batch. Autosampler carryover was evaluated by sequentially injecting the extracted blank plasma → ULOQ sample → two extracted blank plasma samples → LLOQ sample → extracted blank plasma at the start and end of each batch. Selectivity of the method was assessed for potential matrix interferences in six batches of blank rat plasma by extraction and inspection of the resulting chromatograms for interfering peaks. Cross-talk of selective reaction monitoring for analytes and IS was checked using the highest standard on the calibration curve and working solution of IS. Ten non-zero concentrations were used to determine linearity. A quadratic, 1/x2, least-squares regression algorithm was used to plot the peak area ratio (analyte/IS) from selective reaction monitoring versus concentration. Linear equations were used to calculate the predicted concentrations in all samples within the analytical runs. The correlation coefficient for each calibration curve was set at ≥0.998 for both of the analytes. Re-injection reproducibility for the extracted samples was checked by injection of an entire analytical run after storage at 10°C. Intraday accuracy and precision were evaluated by replicate analysis of plasma samples on the same day. The analytical run consisted of a calibration curve and four replicates of the LLOQ, LQC, MQC, and HQC samples. Interday accuracy and precision were assessed by analysis of three precision and accuracy batches on three consecutive validation days. Precision (% CV) at each concentration level from the nominal concentration was set at < 15%. Similarly, values for the mean accuracy were set at 85-115%, except for the LLOQ where the allowed range was 80–120% of the nominal concentration. Stability results in plasma were evaluated by measuring the area ratio response (analyte/IS) of stability samples against freshly prepared comparison standards with identical concentration. Autosampler (wet extract), benchtop (at room temperature), freeze–thaw (at −70°C), and long-term stability (at −70°C) were performed at the LQC and HQC levels using four replicates. Stability data were acceptable if the % CV of the replicate determinations did not exceed 15% and the mean accuracy value was within ±15% of the nominal value. To demonstrate the dilution integrity of the analyte, a pre-determined aliquot was diluted with rat plasma (1:4 and 1:8) and analyzed.

## Results and Discussion

### Mass Spectrometry

**Fig. 2:**
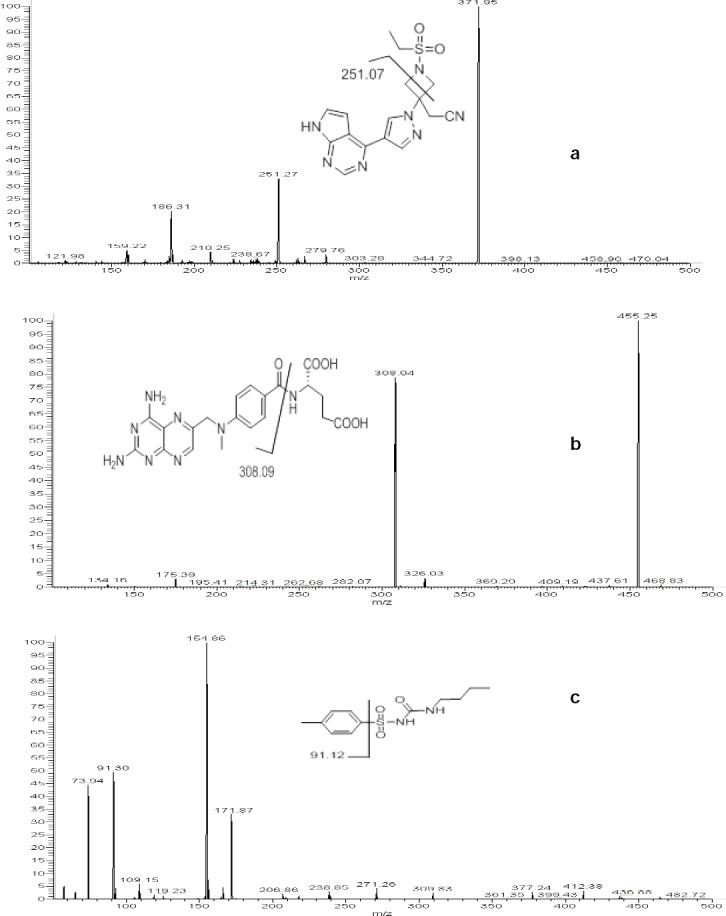
Fragmentation pattern and product ion spectra of (a) baricitinib, (b) methotrexate, and (c) tolbutamide

To determine the most sensitive ionization mode for the components studied, ESI positive and negative were tested with various combinations of components of the mobile phase, i.e. methanol/acetonitrile and water/2 mM ammonium acetate buffer/0.1% formic acid. Signal intensity for [M+H]+ ions in ESI positive ion mode were five-fold higher for all components analyzed using methanol: ammonium acetate buffer compared to experiments run with ESI negative ion mode. Precursor and product ions were optimized by infusing 100 ng/mL solutions in the mass spectrometer between the m/z 100-500 range. The Q1 MS full scan spectra for both the analytes and IS predominantly contained protonated precursor [M+H]+ ions at m/z 372.2, 455.2, and 271.1 for baricitinib, methotrexate, and tolbutamide, respectively. The most abundant and consistent product ions in the product ion spectra were observed at m/z 251.1, 308.1, and 91.1 for baricitinib, methotrexate, and tolbutamide by applying 29, 20, and 34 eV of collision energy, respectively (Fig. 2).

### Liquid Chromatography

**Fig. 3:**
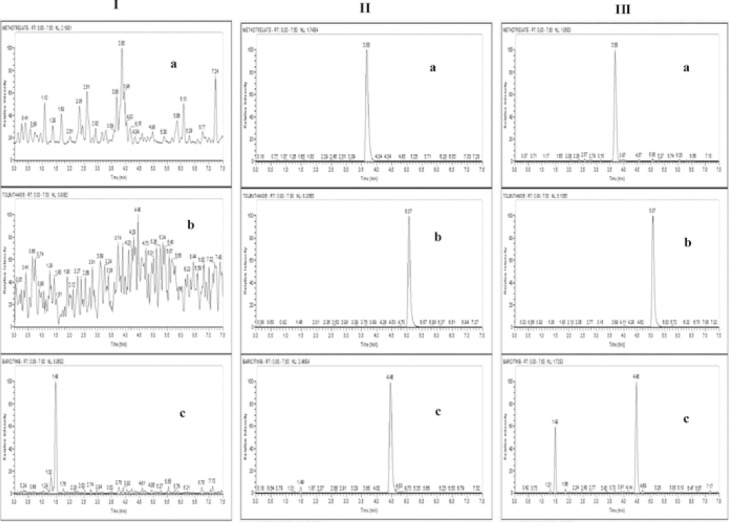
Representative chromatograms for (a) baricitinib, (b) methotrexate, and (c) tolbutamide in the (I) extracted blank plasma, (II) extracted LOQ, and (III) extracted rat pharmacokinetic sample at 1 hr.

Methanol, rather than acetonitrile, was chosen as the organic modifier due to its high sensitivity, better peak shape, and resolution. Ammonium acetate buffer (2 mM) was required to achieve acceptable peak width, shapes, and acceptable ionisation. Samples were run using a reversed-phase C18 column (150 mm × 4.6 mm i.d., 5 µm) (YMC-PACK®, Japan) with 2 mM ammonium acetate: methanol in a gradient mode. All components eluted between 3.5–5.3 min. Representative chromatograms of the extracted blank rat plasma, blank plasma with analytical standards and IS, and rat plasma sample 1 hr after a single-dose administration are shown in Fig. 3.

### Calibration Standard Accuracy and Precision, LLOQ, and LOD

The three calibration curves were linear over the concentration range of 0.5–250.0 ng/mL for baricitinib and methotrexate, with a correlation coefficient (r2) ≥ 0.9970 for both of the analytes. Mean linear equations obtained for baricitinib and methotrexate were y = (0.0153 ± 0.0008) x + (0.00016 ± 0.00012) and y = (0.0141 ± 0.0011) x + (−0.0005 ± 0.00023), respectively. Accuracy and precision (% CV) for the calibration curve standards ranged from 92.6 to 102.6% and 0.6 to 8.8% for baricitinib, and 96.4 to 106.2% and 0.7 to 10.5% for methotrexate, respectively. The lower limit of quantitation (S/N ≥ 20) and limit of detection (LOD, S/N ≥ 5) were 0.5 ng/mL and 0.3 ng/mL for baricitinib and methotrexate, respectively.

### Intra- and Inter-batch Accuracy and Precision

Intra-batch and inter-batch precision and accuracy were established from validation runs performed at four QC levels (Table 2). The within-batch precision (% CV) ranged from 0.9 to 11.6 for baricitinib, 2.7 to 10.2 for methotrexate, while the accuracy was within 93.0–100.5% for baricitinib and 89.1–99.5% for methotrexate. Similarly, for the between-batch experiments, the precision varied from 1.8 to 6.2 for baricitinib and 2.5 to 6.6 for methotrexate, while the accuracy was within 93.0–99.9% for baricitinib, and 95.8–98.8% for methotrexate.

**Tab. 2:**
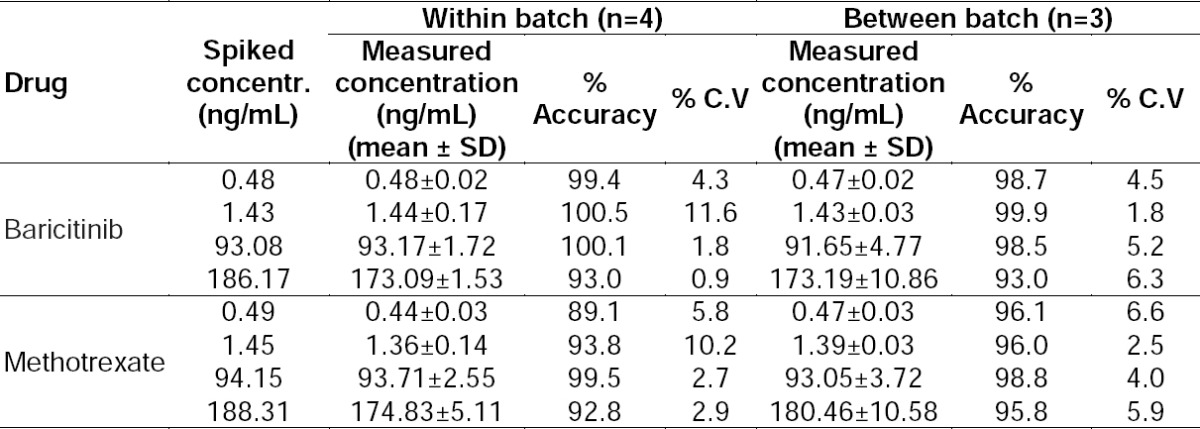
Summary of precision and accuracy from QC samples in Wistar rat plasma

### Stability Results and Dilution Reliability

Stock solutions of analytes and IS for short-term stability tests were stable at room temperature up to 6 h, respectively. Analytes in the control rat plasma (benchtop) were stable for 6 h at 25°C. Analytes in the control rat plasma were stable for three freeze-thaw cycles. Extracted quality control samples were stable up to 24 h at 10°C. Long-term stability of the spiked quality control samples was unaffected up to 30 days at -70°C. Detailed results for stability experiments are presented in Table 3. Precision (% CV) values for reliability of 1/4 and 1/8th dilution were between 2.0–3.0% and 2.7–2.9% for baricitinib and methotrexate, while the accuracy results were within 96.4–99.0%, and 92.8–99.5% for baricitinib and methotrexate, respectively. Results were within the acceptance limit of 15% for precision (% CV) and 85–115% for accuracy as shown in Table 4.

**Tab. 3:**
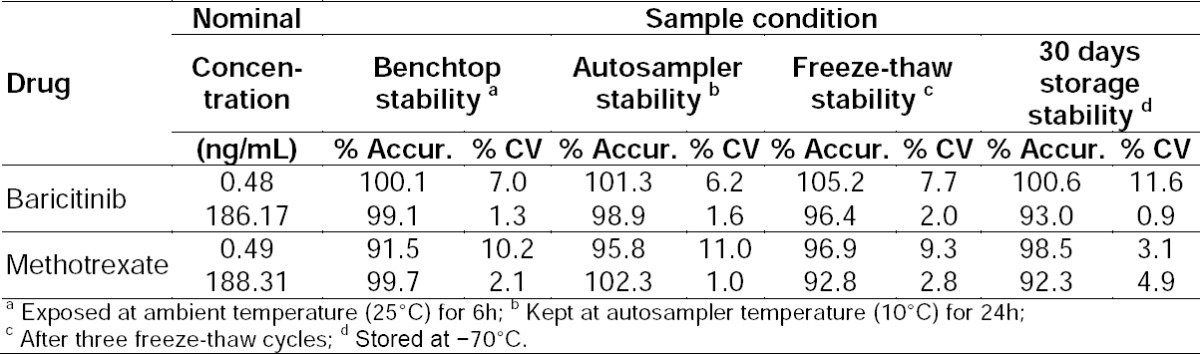
Stability in rat plasma (n=4)

**Tab. 4:**
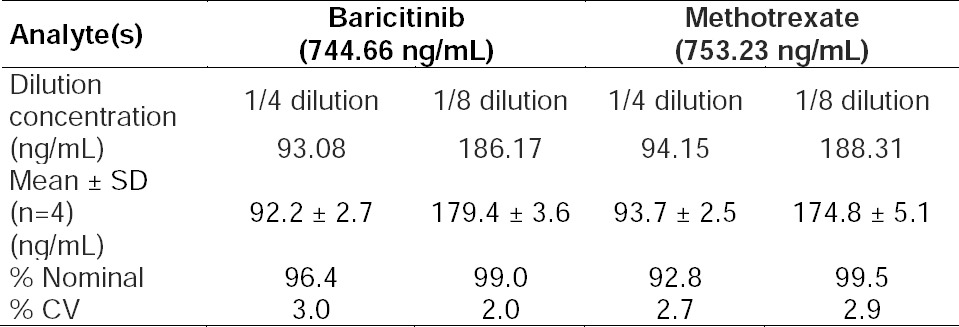
Dilution integrity evaluation of baricitinib and metotrexate in Wistar rat plasma

### Recovery & Matrix Effect

Recoveries of baricitinib and methotrexate from plasma were estimated at their respective low, medium, and high quality control levels. Plasma samples (in quadruplets) containing all analytes at quality control concentrations were also spiked with respective internal standards. Results comparing the peak responses of the post-extraction, spiked samples with those of the pure standards prepared in acetonitrile: water (50:50, v/v) for low, medium, and high quality control levels, indicated that the ratios of the peak responses were within acceptable limits. Absolute recoveries ranged from 85.5 to 88.5% and 92.7 to 86.7% for baricitinib and methotrexate, respectively. The recovery of IS at 125 ng/mL was 91.8% (Table 5). The matrix effect was determined by comparing the analyte and internal standard area ratios of the extracted QC in matrix with the analyte and internal standard area ratios obtained from the neat solution prepared at similar concentration levels [13]. Percent CV of the area ratios at low, medium, and high quality control levels were less than 15% across the analytes.

**Tab. 5:**
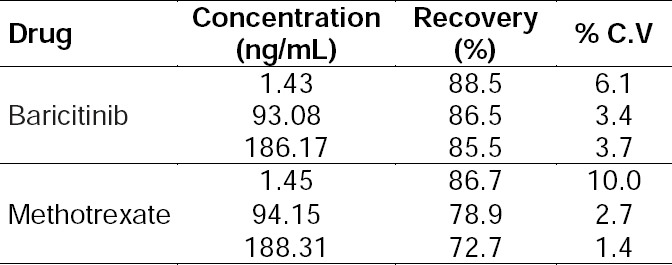
Extraction recovery in rat plasma (n=4)

### Application of the Method in the Pharmacokinetic Study and Incurred Sample Analysis

**Fig. 4:**
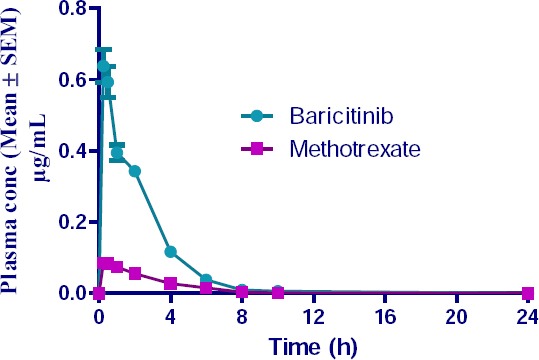
Mean plasma concentration vs. time after single-dose oral administration of baricitinib and methotrexate in six Wistar rats.

Healthy 6–8 week-old female Wistar rats weighing 180 ± 30 g were obtained from Mahaveera Enterprises, Hyderabad and housed at Incozen Therapeutics Pvt. Ltd., Hyderabad in appropriate cages. Animals were maintained under standard laboratory conditions with a regular 12 h day-night cycle in well-ventilated rooms with an average temperature of 24–27°C and relative humidity of 40–60%. Standard pellet laboratory chow diet (Provimi Animal Nutrition India Pvt. Ltd., Bengaluru, India) and water were allowed ad libitum to rats. The study protocol was approved by the Institutional Animal Ethics and Care Committee at Incozen. All applicable national and international ethical guidelines for maintenance and experimental studies with Wistar rats were followed. Oral formulations were prepared in suspension form by triturating an accurately weighed amount of powdered compound in methyl cellulose (0.5%, w/v water) in a gravimetric dilution pattern. Oral doses of 5 and 2 mg/kg were administered using a gavage needle at 5 mL/kg to rats after an overnight fast (12 hr). Feed was offered 4 h after dosing. Blood samples (0.15 mL) were collected from the retro-orbital sinus at pre-dose, 0.25, 0.5, 1, 2, 4, 6, 8, 10, and 24 h post-dose in K2-EDTA (dipotassium ethylenediaminetetraacetic acid) tubes and were kept on ice till further processing. Plasma was separated by centrifugation at 4°C for 10 min at 4000 rpm and stored at −70°C till further analysis. Pharmacokinetic parameters such as the maximum plasma concentration (Cmax), area under the concentration-time curve (AUC), time to reach the maximum concentration (Tmax), half-life (t1/2), and elimination constant (Kel) were estimated by means of a non-compartmental analysis using Phoenix WinNonlin (Pharsight Inc., USA, version 6.1). Statistical parameters like mean, standard deviation, and % CV were calculated by using MS-Excel 2007 (Microsoft®). The described analytical method was applied to generate the plasma concentration versus time profile of baricitinib and methotrexate in plasma following oral administration of baricitinib and methotrexate. Absorption was rapid and with maximum plasma concentrations of 0.60 and 0.10 µg/mL at 0.25 and 0.56 hr after oral administration of baricitinib and methotrexate, respectively. Absorbed baricitinib and methotrexate were eliminated with a half-life of 2.57 and 1.94 hr, respectively. The areas under the plasma curve (AUC0-24) were 1.54 and 0.31 µg.h/mL for baricitinib and methotrexate, respectively. Plasma concentrations were observed up to 10.0 hr for both baricitinib and methotrexate after oral administration of the combination (Fig. 4). Pharmacokinetic parameters of baricitinib and methotrexate are presented in Table 6. In the current study, ISR was performed on 18 plasma samples from six different rats at Cmin, Cmax, and the time point covering the phase of elimination. As per the acceptance criterion, at least two-thirds of the original results and repeat results should be within 20% (Table 7). Data demonstrated the adaptability and successful translation of the validated analytical method for estimation of baricitinib and methotrexate to an *in vivo* setting.

**Tab. 6:**
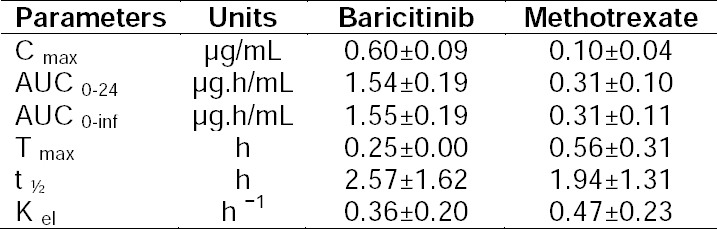
Pharmacokinetic parameters (mean ± S.D.) after single-dose oral administration of baricitinib and methotrexate simultaneously in Wistar rats

**Tab. 7:**
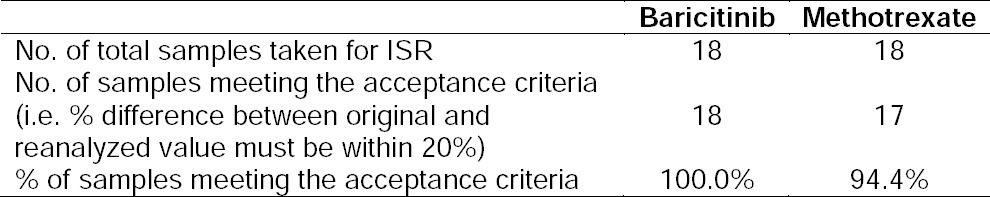
Incurred sample reanalysis of baricitinib and methotrexate

### Comparison with Reported Methods

Available methods for the estimation of methotrexate are based on the use of HPLC-UV detection and a few LC-MS/MS-based assays are also reported [9–12]. Published methods indicate longer run times with a high plasma volume requirement besides issues with low sensitivity. The aim of the present investigation was to develop and validate a simple LC-MS/MS method using a gradient mode with sufficient accuracy and precision for simultaneous estimation of baricitinib and methotrexate and its subsequent use in pharmacokinetic studies in rats. The present method involves a simple protein precipitation procedure with good sensitivity and a gradient reversed-phase LC-MS/MS analysis for all analytes of interest. This method is specific for baricitinib and methotrexate with good linearity, accuracy, and precision. This method involves 50 µL of plasma followed by a single-step protein precipitation extraction procedure. Furthermore, this one-step protein precipitation extraction procedure decreases both the cost and duration of the assay. The chromatographic conditions of this method were optimized for a 7.5 min run time on LC-MS/MS.

## Conclusion

The developed LC-MS/MS method for the quantitation of baricitinib and methotrexate in rat plasma was fully validated as per USFDA guidelines. The proposed method has a much higher sensitivity for both of the analytes compared to other reported methods either as a single analyte or with a combination in different biological matrices. The efficiency of protein precipitation extraction and chromatographic run time of 7.5 min per sample renders the method useful in high-throughput bioanalysis. The absence of matrix interference is effectively shown by the precision (% CV) values for the calculated slopes of calibration curves in different plasma sources. The validated method showed acceptable data for all of the validation parameters, with adequate sensitivity and selectivity for their simultaneous quantification in a clinical setting. Moreover, this is the first combination method for the estimation of baricitinib and methotrexate in rat plasma. Further, incurred sample reanalysis of 18 samples authenticates the reproducibility of the proposed method.
